# Sex and sport: chlamydia screening in rural sporting clubs

**DOI:** 10.1186/1471-2334-9-73

**Published:** 2009-05-27

**Authors:** Fabian YS Kong, Jane S Hocking, Chris K Link, Marcus Y Chen, Margaret E Hellard

**Affiliations:** 1 Centre for Population Health, The Macfarlane Burnet Institute for Medical Research and Public Health, 85 Commercial Rd, Melbourne, Victoria 3004, Australia; 2 Key Centre for Women's Health in Society, University of Melbourne, 305 Cardigan Street, Carlton, Victoria 3053, Australia; 3 Women's Health Loddon Mallee, 47 Myers Street Bendigo, Victoria 3550, Australia; 4 Melbourne Sexual Health Centre, 580 Swanston Street, Carlton, Victoria 3053, Australia; 5 Department of Epidemiology and Preventive Medicine, Monash University, 89 Commercial Rd, Melbourne, Victoria 3004, Australia

## Abstract

**Background:**

Chlamydia trachomatis is the most common notifiable disease in Australia, mainly affecting those aged 15 to 29 years. Testing rates are low in Australia and considerably lower in rural areas, with access and confidentiality of sexual health services being problematic in rural and regional areas. This study aimed to determine the feasibility of establishing a pilot chlamydia testing outreach program among 16–25 year old males and females in rural Victoria (Australia) undertaken at local sporting clubs and to determine the prevalence of chlamydia and acceptability of the program in this population.

**Methods:**

We aimed to recruit young people from the Loddon Mallee region of Victoria, Australia between May and September 2007. After a night of sporting practice, participants provided a first pass urine sample, completed a brief questionnaire regarding risk taking behaviour and were then provided with condoms and health promotion materials about sexually transmitted infections (STIs). Those positive for chlamydia were managed by telephone consultation with a practitioner from Melbourne Sexual Health Centre.

**Results:**

A total of 709 young people participated (77% male, 23% female), 77% being sexually active. All provided a urine sample and completed the questionnaire. Participation rate on recruitment nights was over 95%. Overall chlamydia prevalence in those sexually active was 5.1% (95%CI: 3.4–7.3), 7.4% in females (95%CI: 3.5–13.6) and 4.5% in males (95%CI: 2.7–6.9).

**Conclusion:**

Sporting clubs represent a feasible, acceptable and innovative community based setting to screen, treat and educate young people in a rural and regional setting, especially for males.

## 

Chlamydia (*Chlamydia trachomatis*) is the most prevalent bacterial sexually transmitted infections (STI) in the western world [1] and the most common notifiable infectious disease in Australia. [2] The number of chlamydia notifications in Australia has increased by 92% over the past five years. [3] Infection is largely concentrated among young adults with approximately 80% of notifications being among young men and women aged 15 to 29 years. Chlamydia infection can cause significant morbidity, particularly for women; up to two-thirds of cases of tubal infertility and one-third of cases of ectopic pregnancy may be directly attributable to chlamydia infection.[4] As over 80% of infections in men and women are asymptomatic [4] screening or testing of asymptomatic individuals is necessary to detect cases and provide treatment.

Nationally only about 8% of young women and 2% of young men aged 15 to 24 years are tested for chlamydia each year, mainly through general practitioners [5], with Victorian chlamydia testing data showing that testing rates are considerably lower in rural areas. [6,7] Young people report various difficulties in accessing appropriate health services in rural and regional areas; high visibility in small towns makes it difficult to seek advice, purchase contraceptives and access abortion services, and there is concern about the maintenance of confidentiality by health service providers (including pharmacies) as well as a lack of transport to access services. [8,9] These issues highlight the need for innovative youth friendly screening programs to increase detection and provide access to treatment and education about chlamydia in the community.

We present the results of the "Sex and Sport" project. This project investigated the feasibility of establishing a chlamydia screening and treatment program targeting under-25-year-old-men and women, based in local community sporting clubs in rural areas of the State of Victoria, Australia. The project was undertaken in the Loddon Mallee Region of Victoria, Australia, which covers a geographic area representing 26% of the area of Victoria and 6% of the population. It is estimated that approximately 80% of people aged over 15 years in the Loddon Mallee region participate in exercise, recreation and sport. [10]

## Methods

### Participants

The study was conducted between May and September 2007. Eligible men and women were recruited from local community sporting clubs in the Loddon Mallee region of Victoria. All potential clubs were identified and contacted by telephone or email using local contacts. Clubs agreeing to participate were visited two weeks prior to recruitment so that the project could be explained to the players, information could be provided and questions about the study answered. The use of media (e.g. radio, local newspaper) was used as it encouraged non-participating clubs and clubs yet to be recruited, in the same region, to participate. This technique "exploited" the competitive nature of sporting clubs i.e., if one club was seen to be involved in an interesting project, this created interest in participation in another club, especially given the benefits that participation provided to its members (chlamydia screening and health promotion). Club presidents and respected members of the club (eg. sports coaches) were targeted to deliver the project's benefits to young people with the aim of improving recruitment success. Recruitment was conducted in the club rooms following a training session. All players attending the training night were approached by study researchers and asked if they would like to participate. Two nurses were employed to undertake the recruitment (one in each of the two study regions). These nurses were embedded into an existing community based organisation.

Men and women were eligible if they were aged 16 to 25 years and had sufficient English skills to give informed consent. After providing written informed consent, players completed a brief questionnaire that asked about sexual activity, knowledge and history of STIs, alcohol and drug use, and provided a self-collected, first pass urine specimen. Participants were then provided with a "show bag" with condoms and educational material about STIs and available sexual health services. Participating clubs were provided with refreshments for all club members after training.

#### Urine testing

Urine samples were stored in an ice-brick-cooled, insulated container and transported within 24 hours of collection to the local laboratory and tested for Chlamydia trachomatis using Nucleic Acid Amplification technology (Aptima Combo 2 Assay™, Gen-Probe Inc, USA or BD ProbeTec ET System™, Becton Dickinson, USA). Funding for tests was drawn from the study budget.

#### Provision of results and treatment

Participants with negative results for chlamydia were informed by standard mobile phone short messaging service (SMS; aka text messaging); positive cases were contacted by telephone by a sexual health practitioner, with treatment (Azithromycin 1 g) posted free of charge to the participants in plain envelopes. Management of positive cases was provided in an integrated fashion that enabled participants to be either referred to their local doctor, regional community health/sexual health service or by telephone consultation with Melbourne Sexual Health Centre (MSHC). Positive cases were telephoned three months after treatment to remind them to be retested for chlamydia by their local health care provider.

Ethical approval was provided by the Victorian Department of Health and participants' informed consent was obtained.

### Data

Demographic data, sexual risk behaviour, sexual health knowledge and information relating to drug and alcohol consumption, chlamydia results and participants' contact details were collected. Data was entered into secure Microsoft Access databases.

### Analyses

All data were analysed using STATA version 9 (StataCorp, Texas, USA). Confidence intervals for the chlamydia prevalence and 95% confidence intervals were estimated using exact methods for binomial proportions. Odds ratios and 95% confidence intervals assessing behavioural associations with chlamydia prevalence were calculated using logistic regression. Chi square tests were used to determine the significance of differences between sexes.

## Results

### Demographic details

One hundred and twenty one clubs were approached to participate via a combination of emails and personal contact (48 in the Mallee region and 73 in the Loddon region) with 29 clubs (16 clubs from the Mallee region and 13 clubs from the Loddon region) participating overall, providing a participation rate of 24%. Seven hundred and nine participants were recruited with an overall participation rate of over 95% on the night of recruitment. Five hundred and forty seven (77%) of the participants (121/161 females and 426/548 males) were sexually active, defined as having engaged in vaginal or anal intercourse. Thirty-five (5%) of participants were aged over 25 to 29 years but were included in the analysis as they remained the target population of interest. Only 2% (n = 12) were Indigenous (Aboriginal and/or Torres Strait Islander).

### Chlamydia prevalence

There were 28 positive cases of chlamydia; 19 males and 9 females (one female being indigenous). Twenty (4.5%) of the sexually active participants reported having ever being previously diagnosed with Chlamydia, with only one participant (male) in the study having both previously reported a positive test and tested positive in the study. Of the 28 positive cases for chlamydia, all participants received treatment, 27 (96%) through the study and one (4%) from their local physician.

The overall prevalence among those aged 16–29 years was 3.9% (28/709; 95%CI 2.6, 5.7); 5.6% (9/161; 95%CI 2.6, 10.3) in females and 3.5% (19/548; 95%CI 2.1, 5.4) in males. Among those sexually active the overall prevalence was 5.1% (28/547; 95%CI 3.4, 7.3); 7.4% (9/121; 95%CI 3.5, 13.6) in sexually active females and 4.5% (19/426; 95%CI 2.7, 6.9) in sexually active males. In those sexually active participants, having one new partner in the past three months was associated with a nearly 2.5-fold increase in the odds of infection (OR 2.45, 95%CI 0.99–6.03) and having two or more new partners in the past three months was associated with over three times the odds of infection (OR 3.23, 95%CI 1.23–8.46). Overall, there was a trend towards increased odds of infection with increasing number of sexual partners.

Twenty participants were randomly selected to complete a project evaluation survey. Twelve participants responded, seven male and five female, 11 negative and one positive for chlamydia. Overall the project was considered youth friendly with over 80% reporting the project useful for increasing access to testing, treatment and health promotion, and 92% stating they would be happy to undertake an annual sexual health check at their local sporting club, preferring a telephone consultation and receiving their test results by mobile text messaging.

## Discussion

The major outcome of this study is that chlamydia screening and treatment through sporting clubs is both feasible and acceptable; we obtained a participation rate of over 95% on the night of recruitment. The prevalence of chlamydia in sexually active participants was 5.1%, showing that screening would be cost effective in the population under study. [11]

As mentioned, the study had a high participation rate and user acceptability. The majority of surveyed participants reported they were happy to have an annual sexual health screen at their local sporting clubs with chlamydia results provided by SMS, and had learnt about an STI service of which they had not previously been aware. Integrating the project workers into an existing community based organisation was essential not only to improve user acceptability but to ensure effective use of limited resources in the region and to tap into vital local knowledge on how best to target, recruit and educate young people.

Although it is not was not the primary objective of this study, the population studied was typical of other Australian and overseas studies in regards to both the prevalence of chlamydia and sexual risk behaviour. [12-16] This suggests that the success of our recruitment strategy was not due to our study population being unusual or atypical of young men and women.

Recruiting small clubs that predominantly have very young members (50% of our study sample were aged 17–21 years) may not be cost effective in terms of the detection and treatment of positive cases since many members may not yet be sexually active. Nevertheless, health promotion would still be important in the future prevention of cases, and the intrinsic "community spirit" of local sporting clubs was important for improving participation rate and for the types of clubs identified for recruitment. One organisation that was involved in recruitment (a large private sporting complex) had no regular practice sessions or pre- or post game socialising had the lowest participation rate (40%).

An option for an anonymous, telephone sexual health consultation by telephone such as that provided by MSHC would need to be considered to overcome the high visibility of seeking sexual heath services in rural settings (as informed by the local community health workers and the study Advisory Committee). The cost free aspect of the project was also very attractive to young people due to the low number of free services available in the rural setting and the cost (and visibility) of obtaining treatment. Incentives such as providing meals on the night of recruitment, autographed footballs, lollipops and condoms helped make the project more youth friendly.

Study limitations were that the surveys were often completed in a group setting where participants were able to openly discuss the questions which may have compromised the quality of the completed survey. However this was also an advantage since encouraging a popular person to begin the survey greatly improved participation rates as it was then considered to be acceptable. Recruiting through sporting clubs also reduces the generalisability of our results excluded young people who did not participate in sports. Nevertheless, this effect may be small given 80% of young people in the study region [10] and nationally, 89% of young Australians aged 15–24 years [17] participated in some type of recreation and sport, and the clubs recruited represented the most common sports played in the region (netball and football). The low club participation rate (24%) was predominately due to the final decision about a clubs participation being the responsibility of the club president, secretary or committee rather than the parents or individuals. We have no evidence to suggest there were differences in clubs that participated and those that did not. Anecdotally, on a number of occasions clubs chose not to participate because a committee meeting was required prior to permission being given to the study team to undertake recruitment, and often there was not sufficient time within the recruitment time period to conduct such a meeting. This suggests that if the project continued over a number of years the proportion of clubs participating would increase. Identifying strategies and factors to increase clubs participation in the program would be important prior to the program being implemented on a state-wide level. There were fewer females recruited in the project, mainly due to the lower numbers of players in a netball team compared to a football teams (seven vs 20 players) but of those participating on the night, participation rates were slightly higher in females, most likely as it was seen to be more beneficial to that gender.

However the true population effect of this type of screening is unknown given the limited data on this study population.

## Conclusion

Chlamydia is a significant public health issue that is both easy to test and treat. Our study identified an innovative and feasible screening, treatment and health promotion program in a rural setting, especially for males, who remain an under-targeted population. The project was also highly acceptable to young people and rural health workers. The collaborations developed with the local communities (health services and sporting clubs) increased their capacity to effectively deliver an integrated, community based sexual health service.

## Competing interests

The authors declare that they have no competing interests.

## Authors' contributions

FK was the project officer on the project, who collected, collated and analysed data and prepared the manuscript. JH assisted in the design of the study and was involved in the statistical analysis, interpretation of the data, and drafting of the manuscript. CL assisted in data collection, recruitment of participants and design of the study. MC was involved in the design of the study. MH is the principal investigator who designed the study, assisted in the interpretation of the data and drafting of the manuscript.

## Funding

Australian Department of Health and Ageing Chlamydia Targeted Grants Program as part of a National Chlamydia Pilot program that is currently running to test the effectiveness of a number of models for chlamydia testing in Australia. This project will assist in developing possible recommendations for a National Chlamydia Program. Funding was also received from the Myer Foundation. MH and JH received funding support from the National Health Medical Research Council in Australia.

## Pre-publication history

The pre-publication history for this paper can be accessed here:

http://www.biomedcentral.com/1471-2334/9/73/prepub
